# *In Vitro* Identification and Characterization of CD133^pos^ Cancer Stem-Like Cells in Anaplastic Thyroid Carcinoma Cell Lines

**DOI:** 10.1371/journal.pone.0003544

**Published:** 2008-10-28

**Authors:** Giovanni Zito, Pierina Richiusa, Alessandra Bommarito, Elvira Carissimi, Leonardo Russo, Antonina Coppola, Monica Zerilli, Vito Rodolico, Angela Criscimanna, Marco Amato, Giuseppe Pizzolanti, Aldo Galluzzo, Carla Giordano

**Affiliations:** 1 Laboratory of Molecular Endocrinology, Section of Endocrinology, DOSAC, University of Palermo, Palermo, Italy; 2 Department of Human Pathology, University of Palermo, Palermo, Italy; City of Hope Medical Center and Beckman Research Institute, United States of America

## Abstract

**Background:**

Recent publications suggest that neoplastic initiation and growth are dependent on a small subset of cells, termed cancer stem cells (CSCs). Anaplastic Thyroid Carcinoma (ATC) is a very aggressive solid tumor with poor prognosis, characterized by high dedifferentiation. The existence of CSCs might account for the heterogeneity of ATC lesions. CD133 has been identified as a stem cell marker for normal and cancerous tissues, although its biological function remains unknown.

**Methodology/Principal Findings:**

ATC cell lines ARO, KAT-4, KAT-18 and FRO were analyzed for CD133 expression. Flow cytometry showed CD133^pos^ cells only in ARO and KAT-4 (64±9% and 57±12%, respectively). These data were confirmed by qRT-PCR and immunocytochemistry. ARO and KAT-4 were also positive for fetal marker oncofetal fibronectin and negative for thyrocyte-specific differentiating markers thyroglobulin, thyroperoxidase and sodium/iodide symporter. Sorted ARO/CD133^pos^ cells exhibited higher proliferation, self-renewal, colony-forming ability in comparison with ARO/CD133^neg^. Furthermore, ARO/CD133^pos^ showed levels of thyroid transcription factor TTF-1 similar to the fetal thyroid cell line TAD-2, while the expression in ARO/CD133^neg^ was negligible. The expression of the stem cell marker OCT-4 detected by RT-PCR and flow cytometry was markedly higher in ARO/CD133^pos^ in comparison to ARO/CD133^neg^ cells. The stem cell markers c-KIT and THY-1 were negative. Sensitivity to chemotherapy agents was investigated, showing remarkable resistance to chemotherapy-induced apoptosis in ARO/CD133^pos^ when compared with ARO/CD133^neg^ cells.

**Conclusions/Significance:**

We describe CD133^pos^ cells in ATC cell lines. ARO/CD133^pos^ cells exhibit stem cell-like features - such as high proliferation, self-renewal ability, expression of OCT-4 - and are characterized by higher resistance to chemotherapy. The simultaneous positivity for thyroid specific factor TTF-1 and onfFN suggest they might represent putative thyroid cancer stem-like cells. Our *in vitro* findings might provide new insights for novel therapeutic approaches.

## Introduction

Anaplastic thyroid carcinoma (ATC) is one of the most aggressive endocrine tumors with morphological features of undifferentiated neoplasm. Patients with ATC have a poor prognosis with a mean survival time of 2–6 months. Surgery, radiotherapy and chemotherapy do not improve survival rate [1].

Recently, adult stem cells were identified in human thyroid glands [2]. These cells express several specific markers, such as the nuclear transcription factor OCT-4 (also known as OCT-3, OCT-3/4) and the endodermal markers GATA-4 and HNF4α [2]–[4].

A link between stem and cancer cells has been suggested in various tissues where cancer cells are supposed to derive from immature progenitors or stem cells [5]. Cancer stem cells (CSCs) have been found in leukemia [6], glioblastoma [7], breast [8], prostate [9], gastric [10], lung [11], and colon [12] cancer. These cells, which represent only a small population within the bulk of the tumor, possess the simultaneous ability to self-renew and differentiate into other cytotypes [13]. The stem-like phenotype has proved to be capable of resisting conventional therapies, thus leading to disease relapse even when the primary lesion has been eradicated [14], [15]. To date, however, no studies have definitely indicated that stem cells are responsible for thyroid carcinogenesis. However, the rarity and rapid growth pattern of ATC resembles the nature of stem cells. Only one study has described a very small population, termed side population, enriched for stem cells among thyroid cancer cell lines [16]. In addition, the hypothesis of fetal cell carcinogenesis, in which cancer cells are derived from the remnants of fetal thyroid cells instead of adult thyrocytes, has been proposed [17].

Several markers have been identified for the characterization of CSCs. Human CD133, a highly conserved antigen homologue of mouse Prominin-1, was originally identified in a subpopulation of CD34^+^ hematopoietic cells derived from human fetal liver and bone marrow [18]–[19]. CD133 has been used for the identification and isolation of a putative CSC population from several human cancers [20], [21]. In addition, the expression of CD133^pos^ CSCs in hepatocellular carcinoma (HCC) was shown to confer chemoresistance *in vitro* [14]. However, its biological function remains unknown. The transcription factor OCT-4 is considered a main regulator of human embryonic stem cell pluripotency and self-renewal capacities [22]. Interestingly, these stem-cell properties are attributed to OCT-4A, a splice variant of the OCT-4 gene located in the nucleus [23], [24].

The aim of the present study was to investigate the expression of putative stem cell markers in established human ATC cell lines, such as ARO, KAT-4, KAT-18 and FRO. We identified CD133^pos^ cells in ARO and KAT-4 cell lines. This subset was characterized by higher *in-vitro* proliferation, self-renewal and colony forming ability. ARO/CD133^pos^ were more resistant than ARO/CD133^neg^ cells to chemotherapy-induced apoptosis. In addition, ARO/CD133^pos^ cells expressed the thyroblast specific transcription factor TTF-1 and the stem cell marker OCT-4, whereas they were negative for the stem cell markers c-Kit and THY-1.

## Materials and Methods

### Cell lines and culture conditions

Human ATC cell lines ARO, KAT-4, KAT-18 and FRO were kindly provided by Prof. A. Fusco, University of Naples, Italy. During expansion phase and for self-renewal assay cells were cultured in RPMI 1640 medium containing 10% heat-inactivated fetal bovine serum (FBS). For all other experiments, cells were cultured in RPMI1640 serum free medium (SFM), supplemented with basic Fibroblast Growth Factor (bFGF, 20 ng/ml; Sigma-Aldrich, St. Louis, MO, USA) and Epidermal Growth Factor (EGF, 20 ng/ml; Sigma-Aldrich) [25].

### Flow Cytometry

The expression of stem cell markers CD133, OCT-4, c-Kit and THY-1 was evaluated by flow cytometry (FACSCalibur, Becton Dickinson, San Jose, CA, USA). For CD133 analysis, cells were first treated with FcR blocking reagent (Miltenyi Biotec, Bergisch Gladbach, Germany) and then incubated in the dark at 4°C for 10 minutes with phycoerythrin (PE)-conjugated mouse IgG2b anti human CD133/2 (clone 293C3, Miltenyi Biotec). For co-staining with c-Kit and THY-1, cells were subsequently incubated with monoclonal mouse IgG1 anti-human c-KIT and mouse IgG1 anti-human THY-1 antibodies (Chemicon International, Temecula, Ca, USA) at 4°C for 30 minutes. Cells were washed twice with PBS and then incubated with fluorescein isothiocyanate (FITC)-conjugated polyclonal goat anti-mouse at 4°C for 30 minutes. For staining with OCT-4, cells were fixed and permeabilized with Cytofix-Cytoperm kit (BD Pharmingen, San Diego, Ca, USA) according to the manufacturer's instructions. Cells were then incubated with monoclonal mouse IgG2b anti-human OCT-3/4 (Santa Cruz Biotechnology, Santa Cruz, Ca, USA) at 4°C for 30 minutes, washed twice with PBS and incubated with phycoerythrin (PE)-conjugated polyclonal goat anti-mouse at 4°C for 30 minutes. The primary antibody recognizes the OCT-4A isoform of the protein (aa 1–134).

Apoptosis was evaluated by caspase 3 assay. Cells were first fixed and permeabilized with Cytofix-Cytoperm kit (BD Pharmingen, San Diego, Ca, USA) according to the manufacturer's instructions. Cells were then incubated with monoclonal IgG rabbit anti-Active Caspase 3 (BD Pharmingen) at room temperature for 20 minutes, washed twice with PBS and then incubated with fluorescein isothiocyanate (FITC)-conjugated polyclonal goat anti-rabbit IgG (Santa Cruz Biotechnology) at room temperature for 20 minutes.

Data were analyzed with CELLQuest Pro software (Becton Dickinson Immunocytometry Systems, San Jose, CA, USA). Gating was implemented based on negative control staining profiles.

### Immunocytochemistry

ARO and KAT-4 cell lines were plated in chamber slides (Lab-Tek, Nunc, Inc. Naperville, USA), allowed to attach for 48 hours and then used for immunocytochemistry. Cells were fixed in 3% H_2_O_2_ in methanol for 10 minutes at room temperature, then washed twice in PBS, blocked with 3% BSA and permeabilized with PBS containing 0.1% Triton X-100 for 10 minutes at room temperature. Cells were incubated with mouse anti-human CD133 (IgG1, clone AC133, Miltenyi Biotec) in blocking buffer for 1 hour at room temperature, then rinsed with PBS. Expression was detected using secondary biotinylated antibodies and streptavidin/horseradish peroxidase. Chromogen 3-amino-9-ethylcarbazole (AEC) substrate was used and slides were counterstained with hematoxylin.

### Sorting of ARO/CD133^pos^ cells

ARO cells were trypsinized and labeled with primary CD133-Biotin and Biotin-Microbeads (Indirect CD133 cell Isolation Kit, Miltenyi Biotec), according to the manufacturer's instructions. After magnetic sorting, cell purity was evaluated by flow cytometry using phycoerythrin (PE)-conjugated anti-human CD133/2 (clone 293C3, Miltenyi Biotec).

### Isolation of total RNA, RT-PCR and qRT-PCR

Total RNA was extracted and purified from cultured KAT-4, KAT-18, FRO and ARO (unsorted, sorted CD133^neg^ and CD133^pos^) cell lines using RNeasy Mini Kit (Qiagen, Milan, Italy), including a digestion step with DNase I set. RNA quantity and quality were assessed by UV spectrophotometry. 2 µg total RNA were reverse transcribed in a volume of 20 µl with Oligo dT primers (Applied Biosystems, Darmstad, Germany) and Stratascript RT (Stratagene, Amsterdam, Netherland), according to the manufacturer's protocol. Thyroglobulin (Tg), thyroperoxidase (TPO), sodium/iodide symporter (NIS), oncofetal fibronectin (onfFN) and OCT-4 expression was analyzed by polymerase chain reaction (PCR) [26]–[27]. 1 µl complementary DNA was added to 50 µl reaction containing 5 µl 10× reaction buffer, 50 mmol/L MgCl_2_, 1 µl dNTPs, 50 pmol sense and antisense primers and 0.5 U Taq Gold DNA polymerase. Reactions were carried out at 95°C for 10 minutes; 35 cycles at 95°C for 45 seconds, 55°C for 45 seconds (primer specific) and 72°C for 45 seconds, followed by an extension at 72°C for 7 minutes and termination at 4°C. Primer pair sequences, cDNA fragment sizes and annealing temperatures were as follows: Tg (762 bp): 5′-CTTCGAGTACCAGGTTGATGCC-3′ and 5′-GGTGGTTTCAGTGAAGGTGGAA-3′ (55°C), TPO (593 bp): 5′-TGTGTCCAACGTGTTCTCCACAG-3′ and 5′-AAGACGTGGCTGTTCTCCCAC-3′ (55°C), NIS (179 bp): 5′-CTATGGCCTCAAGTTCCTCT-3′ and 5′-TCGTGGCTACAATGTACTGC-3′ (57°C), onfFN (215 bp): 5′-TCTTCATGGACCAGAGATCT-3′ and 5′-TATGGTCTTGGCTATGCCT-3′ (55°C), OCT-4 (456 bp): 5′-AGCCCTCATTTCACCAGGCC-3′ and 5′-TGGGACTCCTCCGGGTTTTG-3′ (63°C), β-actin (439 bp): 5′-GATGACCCAGATGACCCAGATCATGTTTG-3′ and 5′-AGGCTGGAAGAGTGCCTCA-3′ (55°C). For OCT-4 analysis, nTERA2 cDNA (positive control) was kindly provided by Dr. S. Liedtke, University of Dusseldorf, Germany. For onfFN and TTF-1, TAD-2 cDNA was used as positive control. CD133 and TTF-1 expression was analyzed by real-time quantitative PCR (qRT-PCR) in individual samples. Total 2 µg were used to measure mRNA levels relative to β-actin mRNA expression. PCR primers and probes were purchased from Qiagen (Quantitect Primer Prominin1 and TTF1). All reactions were performed in a final volume of 20 µl with 2 µl cDNA template using a LightCycler (Roche Diagnostics GmbH, Germany). Data analysis was performed with qBASE Browser which employs a Δ-Ct relative quantification model with PCR efficiency correction and single reference gene normalization (β-actin: 5′-GGACTTCGAGCAAGAGATGG-3′, and 5′-AGCACTGTGTTGGCGTACAG-3′) [28].

### *In Vitro* Culture and Cell Proliferation Assay of ARO/CD133^pos^ cells

After isolation, ARO/CD133^pos^ and ARO/CD133^neg^ cells were cultured immediately in SFM supplemented with bFGF and EGF in an atmosphere of 5% CO_2_ at 37°C.

Cell proliferation was assessed by colorimetric assay using 3-(4,5-Dimethylthiazol-2-yl)-2,5-diphenyltetrazolium bromide (MTT) and BrdU (colorimetric) kit (Roche Diagnostics GmbH, Germany). For MTT assay, sorted ARO/CD133^pos^ and ARO/CD133^neg^ cells were plated in a 96-well plate in 100 µl SFM/well and cultured up to 72 hours. Cells were incubated for 4 hours at 37°C with MTT; after incubation, medium was removed and cells were treated with DMSO for 5 minutes. Viability was evaluated by UV absorption spectrum at 550 nm with Microplate Reader Model 550 (Bio-Rad, Richmond, CA, USA).

For BrdU assay, sorted ARO/CD133^pos^ and ARO/CD133^neg^ were plated in a 96-well plate in 100 µl SFM/well and cultured up to 144 hours. Viability was evaluated by UV absorption spectrum at 450 nm with Microplate Reader Model 550 (Bio-Rad, Richmond, CA, USA).

### Self-Renewal Assay of ARO/CD133^pos^ cells

Sorted ARO/CD133^pos^ cells were cultured in RPMI 1640 supplemented with 10% FBS. CD133 expression was detected by flow cytometry on days 0, 4, 8 and 11. At the same time points apoptosis was assessed by Annexin V-FITC Apoptosis Detection Kit (BD Pharmingen, San Diego, Ca, USA) following the manufacturer's instructions.

### Colony formation assay of ARO/CD133^pos^ cells

Sorted ARO/CD133^pos^ and ARO/CD133^neg^ cells were cultured in 6-well plates in methylcellulose SFM (HSC-CFU basic medium, Miltenyi Biotec). Plates were maintained at 37°C in a humidified incubator for 2 weeks. The number of colonies was assessed by microscopic counting.

### Chemotherapy agents

ARO/CD133^pos^ and CD133^neg^ cells were cultured in SFM supplemented with bFGF (20 ng/ml) and EGF (20 ng/ml) with or without cisplatin (5, 10, 15 and 20 µM; Pharma, Austria), doxorubicin (0.5, 1, 1.5 and 2 µM; Ebewe Pharma, Austria), etoposide (10, 30, 100 µM; Teva Pharma, Netherland). The percentage of viable ARO/CD133^pos^ and CD133^neg^ cells was evaluated at 24, 48 and 72 hours by MTT assay and at 48, 96 and 120 hours by BrdU assay.

For apoptosis evaluation, ARO/CD133^pos^ and CD133^neg^ cells were cultured in 6-well plates and treated with the highest concentration of each drug (20 µM cisplatin, 2 µM doxorubicin and 100 µM etoposide) up to 120 hours. Apoptosis evaluation (caspase 3 assay) was assessed by flow citometry as described above.

### Statistical analysis

Statistical analysis was performed with SPSS v. 11 software for Windows. Data are expressed as mean±SD. Statistical comparisons were based on nonparametric tests and statistical significance was defined at p<0.05. For experiments with chemoterapics, differences in viability of ATC cell lines were calculated with the Mann-Whitney test; p for trend with different drug concentrations was calculated with the W of Kendall test.

## Results

### Identification of CD133^pos^ cells in ATC cell lines

**Figure 1:**
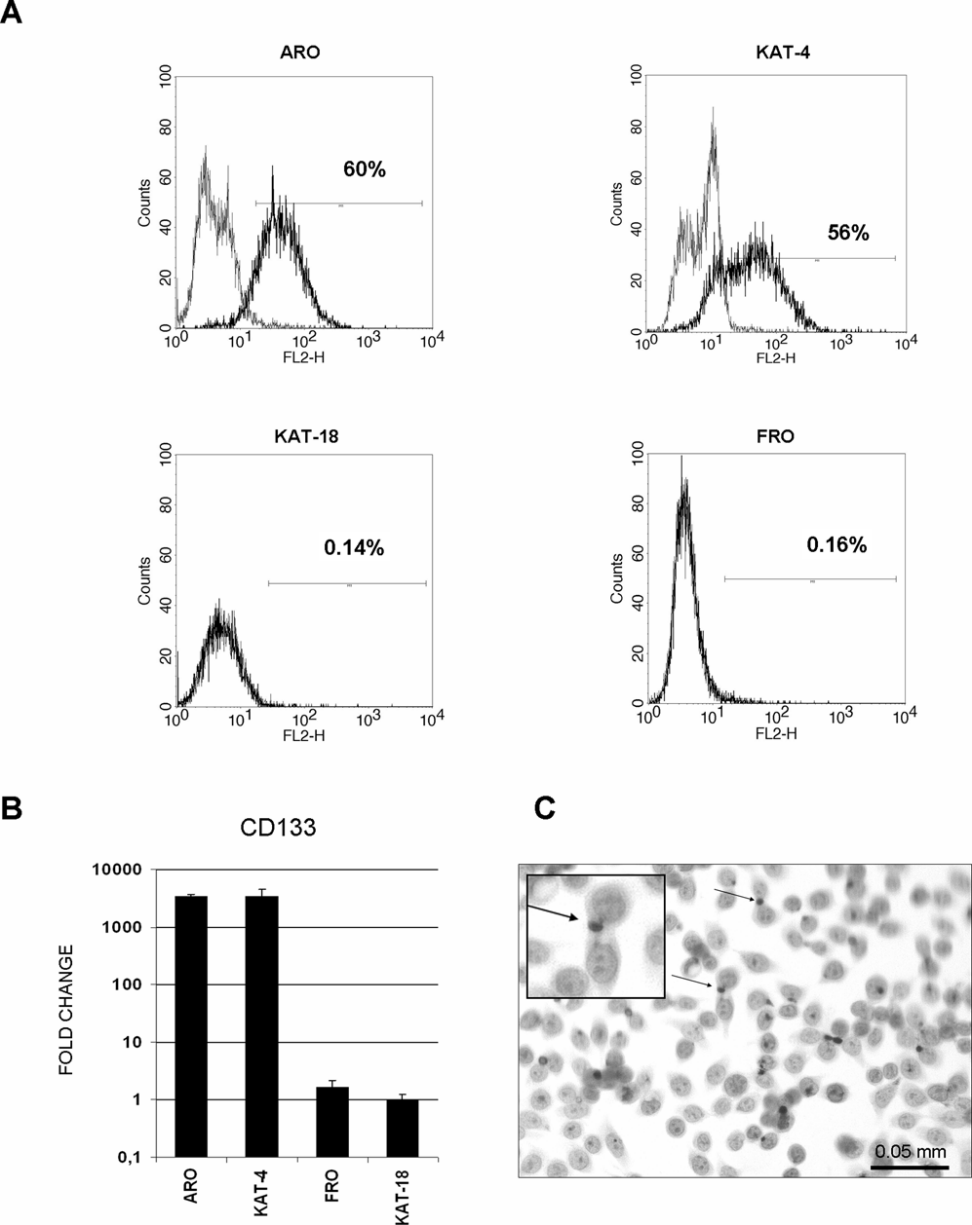
Expression of CD133 in ATC cell lines. (A) Flow cytometry analysis of CD133 in ARO, KAT-4, KAT-18, and FRO cell lines. Black lines represent positive staining for CD133, grey lines show negative control with matched isotype antibody. Data are representative of three independent experiments. (B) Analysis of CD133 expression in ATC cell lines by qRT-PCR. Data are represented as fold change (relative scale), considering KAT-18 = 1. (C) Immunocytochemistry of CD133 in ARO cell line. Arrows indicate apical and polarized localization of CD133 (20× magnification).

**Figure 2:**
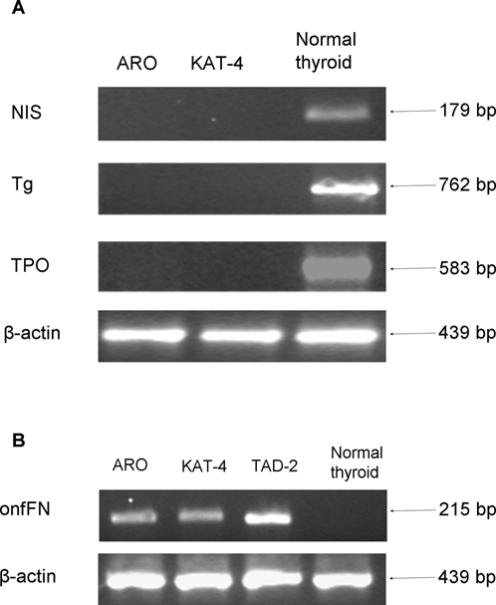
Undifferentiated status of ATC cell lines. Expression of thyroid specific genes (Tg, TPO, NIS) (A) and fetal marker onfFN (B) in ARO, KAT-4 and normal thyroid by RT-PCR. TAD-2 cell line was used as positive control for onfFN mRNA. β-actin was used as internal control in both experiments. bp = base pairs.

In order to identify CSCs in ATC cell lines, we analyzed the expression of CD133 by flow cytometry in ARO, KAT-4, KAT-18 and FRO (Fig 1A). ARO and KAT-4 showed a mean positivity of 64±9% and 57±12%, respectively; KAT-18 and FRO were negative. Results were confirmed by qRT-PCR (Fig. 1B). CD133^pos^ cells in ARO cell lines were characterized by cytoplasmatic and polarized localization of the antigen on the apical surface of the cells (Fig. 1C). The undifferentiated status of ATC cell lines was confirmed in PCR by the presence of onfFN [26] and the absence of thyrocyte-specific differentiating markers Tg, TPO and NIS [27] (Fig. 2A and B).

### *C*ell culture of sorted ARO/CD133^pos^ cells

**Figure 3:**
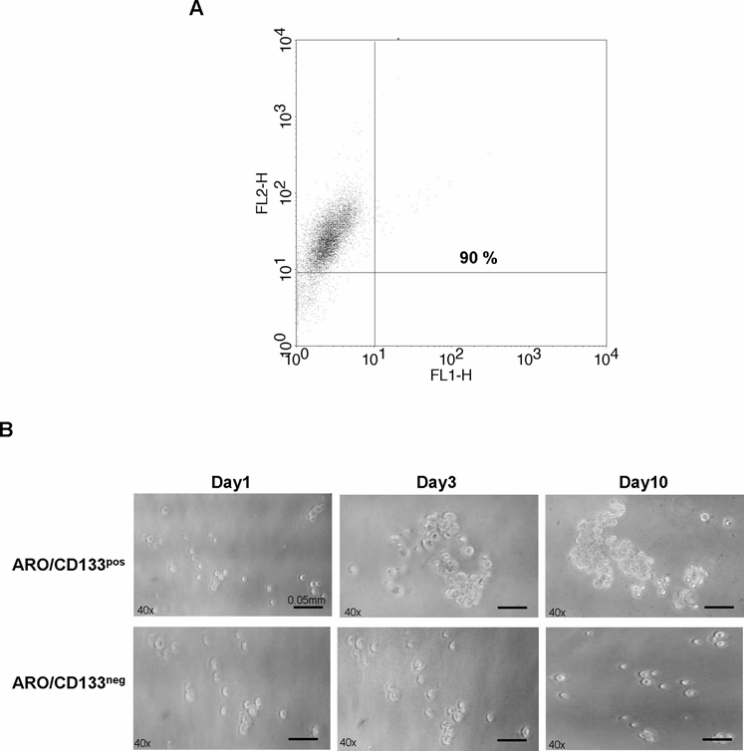
Cluster-forming efficiency of sorted ARO/CD133^pos^ cells. (A) Purity of ARO/CD133^pos^ cells after magnetic cell sorting assessed by flow cytometry. (B) Characteristics of sorted ARO/CD133^pos^ and ARO/CD133^neg^ on days 0, 3 and 10 of culture. ARO/CD133^pos^ cells formed clusters which increased in size overtime.

Cluster-forming efficiency of sorted ARO/CD133^pos^ cells was evaluated by FACS analysis, showing 90% CD133 positivity (Fig. 3A). After isolation, ARO/CD133^pos^ cells, cultured in SFM supplemented with bFGF and EGF, grew as single, non-adherent, spherical cells. After a few days, ARO/CD133^pos^ cells started to form clusters which progressively increased in number and size, although they did not form follicles. On the contrary, ARO/CD133^neg^ cells scarcely aggregated in clusters (Fig. 3B).

### *In vitro* cell proliferation assay, self-renewal and colony-forming capacity of ARO/CD133^pos^ cells

**Figure 4:**
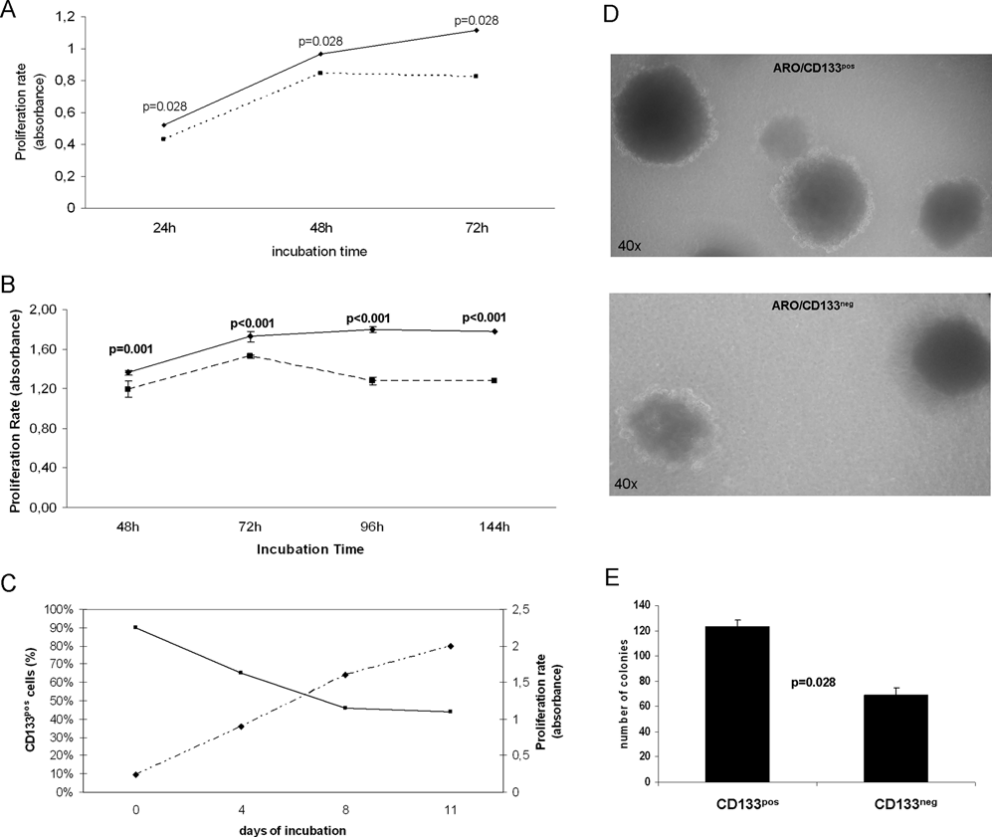
Proliferation, self-renewal and colony-forming capacity of ARO/CD133 cells. (A) Cell proliferation assay (MTT). Dotted line represents ARO/CD133^neg^ cells, continuous line represents ARO/CD133^pos^ cells. Data are expressed as mean values±SD and are representative of four independent experiments. p<0.03. (B) Cell proliferation assay (BrdU). Dotted line represents ARO/CD133^neg^ cells, continuous line represents ARO/CD133^pos^ cells. Data are expressed as mean values±SD and are representative of four independent experiments. p≤0.001. (C) Self-renewal ability of ARO/CD133^pos^ cells. Black line represents number of ARO/CD133^pos^ cells assessed by flow cytometry. Dotted line represents cell viability assessed by MTT assay. (D) Colony formation assay in methylcellulose medium of sorted ARO/CD133^pos^ and CD133^neg^ cells (40× magnification) (E) Number of colonies of ARO/CD133^pos^ and CD133^neg^ cells after 15 days of incubation (p<0.03).

Proliferation was evaluated by MTT and BrdU assay. ARO/CD133^pos^ cells showed *in vitro* increased proliferation in comparison to ARO/CD133^neg^ cells with both MTT at 48–72 hours and BrdU at 72–144 hours (p = 0.028 and p≤0.001, respectively) (Fig. 4A and B). As regards caspase 3, flow cytometry showed spontaneous apoptosis in SFM, which ranged after 144 hours from 0.5 to 2% for ARO/CD133^pos^ cells and from 3.4 to 8.8% for ARO/CD133^neg^ cells (data not shown). Self renewal was also assessed (Fig. 4C). CD133 expression on ARO/CD133^pos^ cells initially decreased from 90% on day 0 to 46% on day 8, and maintained a steady state until day 11. The decrease in CD133 expression overtime was not caused by cell death (<2%, data not shown). Instead, cell proliferation continued for the entire culture period, suggesting that ARO/CD133^pos^ cells are characterized by asymmetrical division ability (i.e. production of two daughter cells, one CD133^pos^ and one CD133^neg^). Colony-forming assay confirmed the high self-renewal ability of ARO/CD133^pos^, in comparison to ARO/CD133^neg^ cells. In fact, ARO/CD133^pos^ cells were able to form a higher number of tumor colonies (p = 0.028) (Fig. 4D and 4E).

### Treatment of ARO/CD133^pos^ and CD133^neg^ cells with chemotherapy drugs

**Figure 5:**
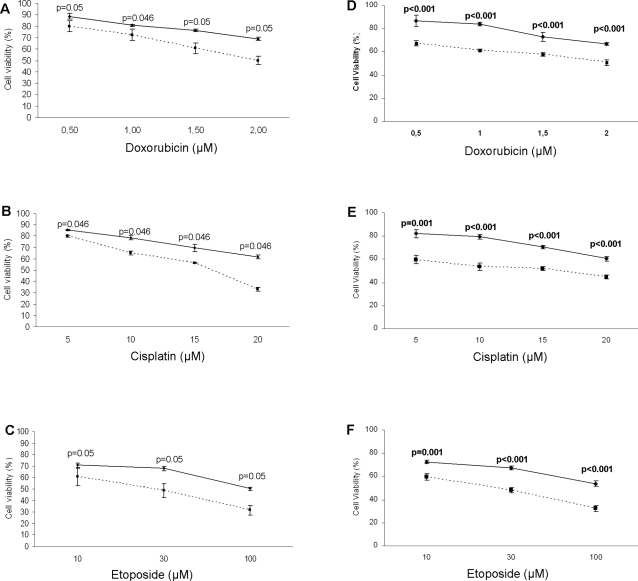
Sensitivity of ARO/CD133^pos^ and ARO/CD133^neg^ cells to chemotherapy agents. (A) MTT analysis after 48 hours of culture in the presence of 0.5, 1, 1.5 and 2 µM doxorubicin (B) after 48 hours in the presence of 5, 10, 15 and 20 µM cisplatin (C) after 48 hours in the presence of 10, 30 and 100 µM etoposide. Data are expressed as mean values±SD and are representative of three independent experiments (p≤0.05). (D) (E) (F) represent BrdU analysis in the same conditions of A, B, C (p≤0.001). Dotted line represents ARO/CD133^neg^ cells and continuous line represents ARO/CD133^pos^ cells in all graphs.

**Figure 6:**
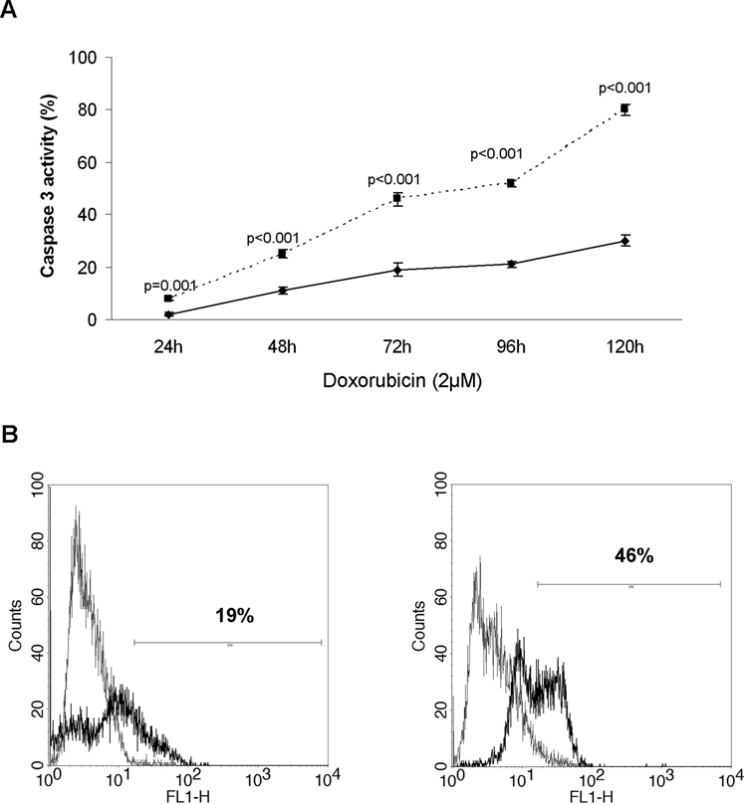
Flow cytometry analysis of caspase 3 in ARO/CD133^pos^ and ARO/CD133^neg^ cells after treatment with chemotherapy agents. (A) Caspase 3 activity after treatment with doxorubicin. Dotted line represents ARO/CD133^neg^ cells, continuous line represents ARO/CD133^pos^ cells. Data are expressed as mean±SD (p≤0.001). (B) Caspase 3 activity at time point 72 hours with doxorubicin. Black line represents positive staining for caspase 3, grey line shows negative control with matched isotype antibody. Data are representative of three independent experiments.

Drug sensitivity was evaluated by MTT and BrdU assay. ARO/CD133^pos^ and ARO/CD133^neg^ cells were incubated with different concentrations of doxorubicin, cisplatin and etoposide. ARO/CD133^pos^ cells showed a remarkable drug resistance to the three agents compared with ARO/CD133^neg^ cells during the entire culture period (p≤0.05 for MTT and p≤0.001 for BrdU, respectively). One representative experiment after 48 hours of each treatment is shown in Fig. 5A–F. Consistently with these findings, apoptosis assessed by caspase 3 activity showed a dramatic activation in ARO/CD133^neg^ in comparison to ARO/CD133^pos^ cells at all time points analyzed (Figure 6A, representative of three independent experiments with doxorubicin). Histograms at time point 72 hours are shown in Figure 6B. Similar results were obtained with the other drugs used (data not shown).

### *In vitro* characterization of ARO/CD133^pos^ cells

**Figure 7:**
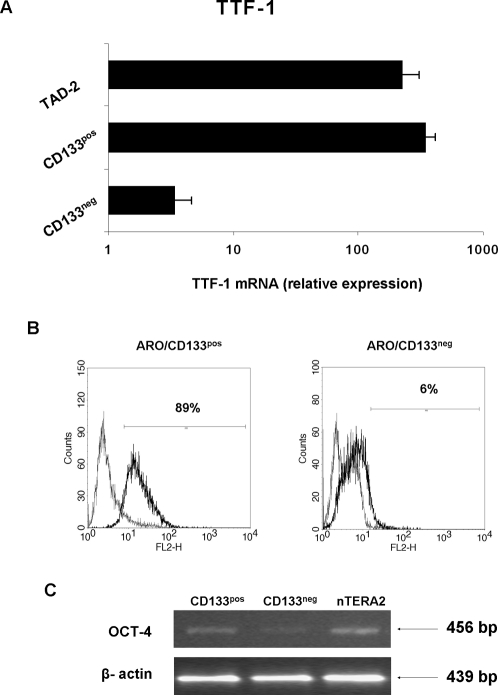
Expression of TTF-1 and OCT-4 in ARO/CD133^pos^ and ARO/CD133^neg^ cells. (A) qRT-PCR of TTF-1 mRNA expression in ARO/CD133^pos^ and CD133^neg^ cells; TAD-2 cell line was used as positive control. (B) Flow cytometry analysis of OCT-4A in ARO/CD133^pos^ and ARO/CD133^neg^ cells. Black line represents positive staining for OCT-4A, grey line shows negative control with matched isotype antibody. (C) Semiquantitative RT-PCR of OCT-4 mRNA in ARO/CD133^pos^ and ARO/CD133^neg^ cells. β-actin was used as internal control. nTERA2 cell line was used as positive control. Data are representative of three independent experiments.

In order to further characterize the ARO/CD133^pos^ population, we evaluated the co-expression of other stem cell markers. The mRNA expression of thyroid transcription factor-1 (TTF-1) in ARO/CD133^pos^ was significantly higher than in ARO/CD133^neg^ cells, similar to TAD-2 fetal thyroid cell line (positive control) (Fig. 7A). OCT-4 expression was 91±3% in ARO/CD133^pos^ cells *vs* 5±1.5% in ARO/CD133^neg^, suggesting the pluripotent stem cell features of ARO/CD133^pos^ cells (Figure 7B). These data were confirmed by semiquantitative PCR, using nTERA2 cell line as positive control (Figure 7C). Quantification, expressed as relative to nTERA2 density, showed OCT-4 mRNA levels in ARO/CD133^neg^ cells almost two fold lower than in ARO/CD133^pos^ (35% *vs* 62% respectively, with nTERA2 = 100%).

The membrane stem cell markers c-Kit - expressed in ESCs (embryonic stem cells) - and THY-1 - typical of mesenchymal and hematopoietic stem cells - were negative (data not shown).

## Discussion

In the last few years several studies have been published supporting the hypothesis that tumors arise from heterogeneous cell populations with different biological properties. Recently, it has been suggested that stem cells, characterized by self-renewal and differentiation ability, may play a role in cancer development [29], [30]. Since multiple mutations occurring over many years are necessary before a cell becomes cancerous, stem cells with a long life-span may be the best candidates for accumulating such cancer-inducing heterogeneous cells [5], [31]. However, it is not clear yet whether or not these CSCs originate from dedifferentiation of mature cells within the organs or from resident stem cells which progressively acquire a malignant phenotype [32].

ATC is one of the most aggressive endocrine tumors characterized by high degree of dedifferentiation [1]. The existence of resident thyroid stem cells may account for both the sustained proliferation and heterogeneity of ATC lesions [4]. Takano et al. hypothesized that a close relationship between stem cells and carcinogenesis might exist in ATC [17], [26], [33]. ATC gene expression profile suggested at least three types of undifferentiated cells as its origin: thyroid stem cells, expressing onfFN mRNA but not Tg, with high differentiation potential, self-renewal and ability to generate anaplastic carcinomas; thyroblasts, expressing both Tg and onfFN mRNA and not forming follicles; prothyrocytes, which are more differentiated than thyroblasts, expressing Tg but not onfFN mRNA, with the ability to form follicles. Mitsutake et al. have identified a very small side population (SP), highly enriched for stem cells, in several human thyroid cancer cell lines. However, both SP^pos^ and SP^neg^ populations formed tumors when transplanted in nude mice, demonstrating that cancer stem-like cells are not exclusive or identical to SP cells [16].

In the present study, we ourselves maintain that ATC may originate from thyroid stem cells. We describe CD133^pos^ cells with phenotypical characteristics of stem cells, growing without forming follicles. However, among the four cell lines analyzed, only ARO and KAT-4 showed high percentage of CD133^pos^ cells (64±9% and 57±12%, respectively; Fig. 1A). Our results are consistent with recent observations on highly aggressive hepatoma cell lines, also characterized by very high percentage of CD133^pos^ cells [34]. High CD133 expression in ATC might be therefore associated with tumor aggressiveness. However, the absence of this marker in KAT-18 and FRO cell lines, suggests that the mere presence of CD133 is not sufficient and other markers still need to be identified. In addition, as reported by Takano et al. [17], we found that ARO and KAT-4 cell lines expressed onfFN but not Tg, TPO and NIS, confirming their undifferentiated status.

To better characterize the functional and phenotypic features of these putative CSCs in ATC, we studied sorted ARO/CD133^pos^ cells. ARO/CD133^pos^ cells showed higher cell proliferation rate in comparison to CD133^neg^ cells (Fig. 4A and B). Furthermore, the self-renewal ability of ARO/CD133^pos^ cells was confirmed by decrease in CD133 expression parallel to increase in cell proliferation, thus suggesting asymmetric division, i.e. production of CD133^pos^ and CD133^neg^ daughter cells. However, mechanisms other than asymmetric division (e.g. CD133 post-transcriptional down-regulation) cannot be excluded. Minimal cell death percentages exclude that the decrease in CD133 expression was due to apoptosis (<2%).

Further confirmation that most of the ARO/CD133^pos^ cells may be stem/progenitor cells comes from expression analysis of other genes related to “stemness”. Although the stem cell markers c-Kit and THY-1 were negative, a strong positivity was found for OCT-4. OCT-4 belongs to the POU (Pit-Oct-Unc) family of transcription factors which mediates pluripotency in ESCs through the inhibition of tissue-specific and promotion of stem-cell genes [22], [23]. ARO/CD133^pos^ sorted cells were strongly positive for OCT-4A in comparison to ARO/CD133^neg^ cells, and their expression was similar to that of nTERA2 embryonic teratoma cell line. The primers and the monoclonal antibody used in our experiments for the nuclear splicing variant OCT-4A exclude any pseudogene contamination or artifacts [24].

The nuclear thyroid specific transcription factor TTF-1 is a homeodomain-containing protein belonging to the Nkx-2 class of homeobox genes, which is required for proper thyroid development and is used as a marker of thyroid and lung carcinoma [35]. TTF-1 expression was found significantly higher in ARO/CD133^pos^ than in ARO/CD133^neg^ cells, with mRNA levels comparable to TAD-2 fetal thyroid cell line (positive control). This implies that although all ATC cell lines are dedifferentiated (absent expression of thyroid-specific genes such as Tg, TPO and NIS and positivity for onfFN), in ARO/CD133^pos^cells a marker of thyroid organogenesis is still maintained.

Furthermore, in order to functionally characterize these putative cancer stem-like cells, we tested sensitivity to the most common chemotherapy drugs used in ATC, i.e. cisplatin, doxorubicin and etoposide. Cisplatin crosslinks DNA in several different ways interfering with cell division by mitosis, doxorubicin interacts with DNA by intercalation and inhibition of macromolecular biosynthesis and etoposide inhibits the enzyme Topoisomerase II [36]–[38]. ARO/CD133^pos^ cells revealed a significant resistance to all drugs used at each time point in comparison to ARO/CD133^neg^ cells, as demonstrated by markedly lower apoptosis levels detected via caspase 3 (Fig. 6). The potential induction of antiapoptotic rather than apoptotic molecules, or the blockage of thyrocyte physiological cell-turnover regulatory mechanisms, demonstrated in other thyroid diseases, such as autoimmune thyroiditis [39], might explain the acquired capability of thyroid stem-like cells to become resistant to chemotherapy. As shown in glioblastomas, a better comprehension of the mechanisms allowing ARO/CD133^pos^ cells to resist conventional therapies may help to find ways to manipulate them to become sensitive to these properties [40].

Our study suggests the existence of cancer stem-like cells in ATCs. However, because there is no unanimous consent about the existence of thyroid stem cells or CSCs in human thyroid, the precise nature of such cells still remains to be established [41].

Schweppe et al. [42] recently questioned the use of thyroid cell lines for identification of molecular aspects involved in carcinogenesis and particularly of ARO 81-1 and KAT-4 cell lines. However, ARO and KAT-4 cell lines used in our experiments, expressed thyroid specific factors TTF-1 and onfFN, confirming their thyroid origin. In addition, PAX8 was also positive (data not shown).

A conclusive answer might derive from *in vivo* experiments in nude mice. However xenograft transplantation by itself may fail to identify true cancer initiating human cells that are not conducive to growth in a non-permissive mouse microenvironment, as recently suggested by Shmelkov et al. [43]. CD133^neg^ cells from metastatic colon cancer were shown to initiate tumors when injected in NOD/SCID mice, challenging the dogma that CD133^pos^ derived from human colon carcinomas are the only cells that could initiate tumor in xenograft models. Indeed, the existence of a high heterogeneity of tumorigenic factors could explain the negativity of KAT-18 and FRO cell lines for CD133. Nevertheless, our preliminary data with immunohistochemical analysis in 5 ATC specimens showed variable positivity for CD133 in all the samples analyzed (personal observations, not published). These results warrant further investigation on large numbers to definitely confirm CD133 expression in all ATC *ex vivo* specimens.

In conclusion, our study may contribute to the elucidation of thyroid carcinogenetic mechanisms in ATC, providing new insights for novel therapeutic approaches.
